# Synapse Maturation and Developmental Impairment in the Medial Nucleus of the Trapezoid Body

**DOI:** 10.3389/fnint.2022.804221

**Published:** 2022-02-09

**Authors:** Sima M. Chokr, Giedre Milinkeviciute, Karina S. Cramer

**Affiliations:** Department of Neurobiology and Behavior, University of California, Irvine, Irvine, CA, United States

**Keywords:** cochlear nucleus, medial nucleus of the trapezoid body, tonotopy, synaptic pruning, calyx of Held

## Abstract

Sound localization requires rapid interpretation of signal speed, intensity, and frequency. Precise neurotransmission of auditory signals relies on specialized auditory brainstem synapses including the calyx of Held, the large encapsulating input to principal neurons in the medial nucleus of the trapezoid body (MNTB). During development, synapses in the MNTB are established, eliminated, and strengthened, thereby forming an excitatory/inhibitory (E/I) synapse profile. However, in neurodevelopmental disorders such as autism spectrum disorder (ASD), E/I neurotransmission is altered, and auditory phenotypes emerge anatomically, molecularly, and functionally. Here we review factors required for normal synapse development in this auditory brainstem pathway and discuss how it is affected by mutations in ASD-linked genes.

## Introduction

Neurodevelopmental disorders with auditory phenotypes, such as autism spectrum disorder (ASD) and schizophrenia (SZ), display altered balance of excitatory and inhibitory (E/I) neurotransmission throughout the brain. Sound localization depends on the E/I ratio in auditory brainstem nuclei and higher auditory structures. Errors in neurotransmission lead to altered signal speed, strength, duration, and ultimately signal interpretation. A consequence of these E/I neurotransmission errors can be observed in ASD, which is often accompanied by sensory symptoms including sound hyper- or hyposensitivity (Van der Molen et al., 2012; Knoth et al., 2014). The establishment of normal E/I ratios in auditory brainstem nuclei begins during embryonic and postnatal development, with additional refinement after hearing onset. Impairments in sound localization have been reported in patients with ASD and SZ (Matthews et al., 2007; Perrin et al., 2010; Visser et al., 2013; Smith et al., 2019), and studies of young and adult brains showed abnormalities in brainstem sizes (Hashimoto et al., 1992; Nopoulos et al., 2001; Claesdotter-Hybbinette et al., 2015). Epidemiological data have highlighted a potential for ASD susceptibility during a gestational period of brainstem development (reviewed in Dadalko and Travers, 2018). Studies using animal models of autism have shown alterations in the E/I ratio and signal strength in the sound localization pathway during postnatal development (Rotschafer et al., 2015; Ruby et al., 2015; Garcia-Pino et al., 2017; Smith et al., 2019). However, physiological differences of auditory brainstem development in sound processing disorders require further investigation. Notably, brainstem alterations in SZ and attention deficit hyperactivity disorder (ADHD) are poorly understood. Here, we discuss factors required for normal development of the E/I ratio in the sound localization circuit and summarize signaling pathways that are altered in models of ASD.

## Medial Nucleus of the Trapezoid Body: Development and Effects of Neurodevelopmental Disorders

**FIGURE 1:**
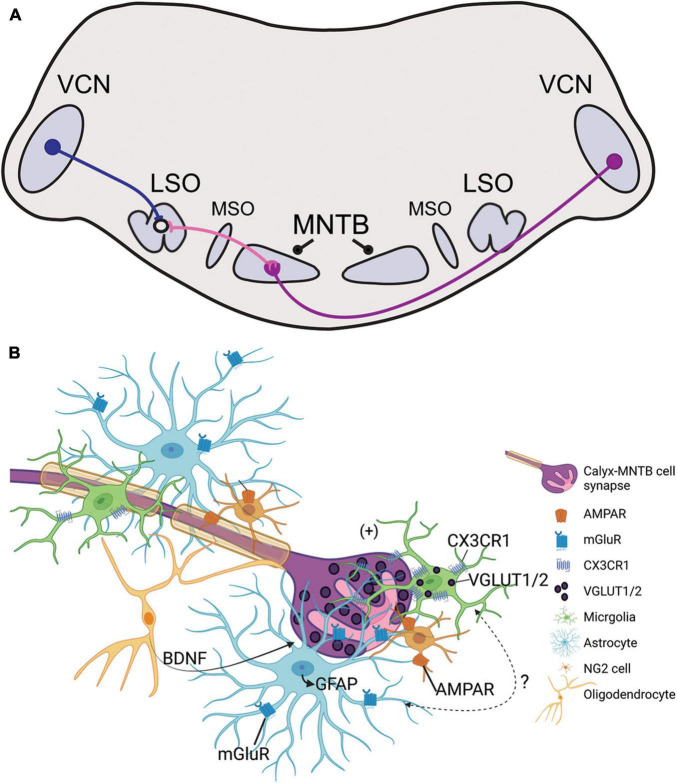
**(A)** Illustration of the sound localization pathway in the auditory brainstem. Globular bushy cells (purple) cross the midline and terminate onto the contralateral medial nucleus of the trapezoid body (MNTB) through the calyx of Held. MNTB neurons provide inhibitory input to cells in the medial superior olive (MSO) and lateral superior olive (LSO; projections shown in pink). LSO neurons simultaneously receive excitatory input from the ipsilateral ventral cochlear nucleus (VCN) via spherical bushy cells (blue). The excitatory/inhibitory ratio in the LSO is used in interaural level difference computation to facilitate sound source localization. **(B)** Schematic representation of glial signaling at the calyx of Held during development. The GBC axon is highly myelinated and terminates in the calyx of Held (purple), which is surrounded by microglia (green), astrocytes (light blue), NG2 cells (orange), and oligodendrocytes (yellow). Glutamatergic vesicles (dark purple) are released from the calyx and dominantly modulate the MNTB neuron (pink). Synapse development and strengthening depend on oligodendrocyte secretion of BDNF, and receptors such as NG2-AMPAR and astrocyte-mGluR which respond to calyceal glutamatergic release. Microglia contain VGLUT1/2 puncta and express CX3CR1, a receptor that modulates inhibitory pruning in the MNTB during circuit formation. Microglia elimination reduces GFAP expression in the MNTB but the signaling mechanism involving microglia-astrocyte communication has not been identified.

Auditory stimuli are detected by cochlear hair cells that transmit signals centrally through peripheral processes of spiral ganglion neurons (SGN). Central processes of SGNs bifurcate upon entering the brainstem to innervate the ventral and dorsal parts of the cochlear nucleus (CN) (Fekete et al., 1984). SGNs directly connect the hair cell in the periphery to its neuronal target in the CN, which then relays excitatory glutamatergic signals to auditory brainstem nuclei and higher auditory structures. Globular bushy cells (GBCs) receive endbulb inputs from SGNs and project to the contralateral medial nucleus of the trapezoid body (MNTB) through a specialized central synapse, the calyx of Held (Figure 1A; Harrison and Irving, 1966; Spirou et al., 2005). MNTB neurons provide inhibitory glycinergic input to the lateral superior olive (LSO), medial superior olive (MSO), ventral nucleus of the lateral lemniscus, and superior periolivary nucleus (SPON) (Liu et al., 2014; Kulesza and Grothe, 2015; Kopp-Scheinpflug et al., 2018; Torres Cadenas et al., 2020). The MNTB is a main contributor of inhibition within the sound localization pathway through its termination onto MSO and LSO neurons and provides monaural temporal information via its connection to the SPON (Zarbin et al., 1981; Moore and Caspary, 1983; Kuwabara and Zook, 1992; Sommer et al., 1993; Behrend et al., 2002; Dehmel et al., 2002; Kulesza, 2007). LSO simultaneously receives excitatory projections from spherical bushy cells in the ipsilateral ventral cochlear nucleus (VCN) and inhibitory input from ipsilateral MNTB (Figure 1A). The balance of excitation and inhibition in LSO allows for computation of interaural level differences used in sound localization. Tonotopy is conserved across the central auditory pathway, which in turn allows the listener to localize sounds based on signal speed, intensity, and frequency within the brainstem and higher auditory regions.

The precision of the sound localization pathway requires orchestrated maturation of cell number, synapse number and strength, and neurotransmitter phenotypes (Kotak et al., 1998; Nabekura et al., 2004; Gillespie et al., 2005; Lee et al., 2016). In the VCN, the endbulb of Held expands and develops elaborate branches (Cant and Morest, 1979; Ryugo and Sento, 1991; Nicol and Walmsley, 2002). The establishment of the mature calyx of Held in MNTB requires the elimination of multiple small inputs until exactly one calyx remains, strengthens, and forms a highly reticulated encapsulation of a principal cell soma by the onset of hearing at about P12 (Held, 1893; Kuwabara and Zook, 1991; Kuwabara et al., 1991; Hoffpauir et al., 2006; Holcomb et al., 2013). As calyces mature, MNTB neurons exhibit faster IPSC depression following hearing onset (Rajaram et al., 2020). MNTB-LSO connections also strengthen as they decrease in IPSC amplitude leading up to hearing onset while MNTB-MSO synapse amplitudes continue to decrease following hearing onset (Kim and Kandler, 2003; Magnusson et al., 2005; Walcher et al., 2011; Pilati et al., 2016; Rajaram et al., 2020). In the gerbil, MNTB-LSO synapse strengthening is largely completed by the third postnatal week, and studies using cochlear ablations suggest that synaptic pruning and topographic establishment during the postnatal period are activity-dependent (Sanes et al., 1992; Sanes and Takács, 1993; Kandler and Gillespie, 2005).

### Factors Required for Proper MNTB Development

The MNTB forms by E17 (Morest, 1969; Kandler and Friauf, 1993; Hoffpauir et al., 2010), and proto-calyceal inputs can be seen before birth (Hoffpauir et al., 2010; Borst and Soria van Hoeve, 2012). Axon guidance molecules such as ephrin-B2, Netrin-1, DCC, and Robo3 guide GBC axons across the midline toward contralateral MNTB (Howell et al., 2007; Hsieh et al., 2010; Yu and Goodrich, 2014). Bone morphogenic protein (BMP)-receptor signaling early in development is required for correct GBC axonal targeting, pruning, and calyceal growth (Kolson et al., 2016b; Kronander et al., 2019). BMP signaling is altered in autism model organisms, and in humans, several signaling pathways associated with BMP are disrupted in ASD (Kumar et al., 2019). For instance, in the rodent, silencing *Fmr1*, a gene linked to fragile X syndrome, which is often correlated with autism, leads to an upregulation in BMP type II receptor and its signaling kinase (Kashima et al., 2016). In the brainstem, *Fmr1* deletion leads to stunted SOC nuclei development, reduced pruning of inhibitory synapses in the MNTB and CN, and delays in auditory brainstem signal propagation (Rotschafer et al., 2015; Ruby et al., 2015; McCullagh et al., 2020). In the LSO, *Fmr1* KO mice showed higher levels of excitatory input strength while inhibitory synapses were not affected (Garcia-Pino et al., 2017). Recent studies have noted hypoplasia in autistic brains, with significant reductions in SOC nuclei size, and cell volume and shape in the MNTB (Kulesza et al., 2011; Lukose et al., 2015). It is thus clear that within the MNTB there are anatomical and molecular abnormalities, impairments in synapse development and elimination, and functional deficits which result from genetic manipulation of an ASD-linked gene.

## Synapse Organization and Strengthening

Proper synapse development in the MNTB requires spontaneous firing patterns, which aid in the establishment of topographic arrangements of cell structure and function along the MNTB mediolateral axis (Hoffpauir et al., 2006; Rodriguez-Contreras et al., 2008; Holcomb et al., 2013; Xiao et al., 2013). In newborn prehearing rodents, spatially restricted and synchronous spontaneous activity in inner hair cells propagates along the developing auditory brainstem and refines the tonotopic maps (Friauf et al., 1999; Kandler et al., 2009; Sonntag et al., 2009; Tritsch et al., 2010; Crins et al., 2011; Leighton and Lohmann, 2016; Sun et al., 2018; Di Guilmi and Rodríguez-Contreras, 2021). Prior to P4, MNTB axons are abundant yet topographically imprecise (Sanes and Siverls, 1991). By P9, MNTB-LSO connections are refined, topographic precision is increased, and synapses are strengthened following activity-dependent pruning (Sanes and Friauf, 2000; Kim and Kandler, 2003; Müller et al., 2009, 2019; Hirtz et al., 2012; Clause et al., 2014). Genetic removal of the α9 subunit of nicotinic acetylcholine receptors (α9 KO) affects spontaneous firing patterns without altering overall activity levels and prohibits functional and structural sharpening of the inhibitory tonotopic map in the projection from MNTB to LSO (Clause et al., 2014), demonstrating that temporal patterns of spontaneous activity are important in development.

The mature MNTB contains a cell size gradient, which increases from the most medial (high frequency) to the most lateral (low frequency) regions (Weatherstone et al., 2017; Milinkeviciute et al., 2021a). *Fmr1* KO mice have a delay in the establishment of the cell size gradient across the MNTB mediolateral axis (Rotschafer et al., 2015). Calyces also increase in size along the tonotopic axis (Milinkeviciute et al., 2021a). Membrane capacitance is correlated with larger synaptic input across the tonotopic axis and time constants are faster in the medial neurons compared to the lateral neurons (Weatherstone et al., 2017). These tonotopic variations reflect the optimization of MNTB cells for function at a wide range of frequencies.

Ion channels are tonotopically distributed in the MNTB, and this gradient can be disrupted with hearing impairment (von Hehn et al., 2004). Kv3.1 tonotopic distribution is lost in mice lacking *Fmr1* (Strumbos et al., 2010). Congenital removal of *Pak1*, an autism-linked gene which normally regulates the development and maintenance of hair cell stereocilia, results in profound hearing loss and a reduction in synapse density in the cochleae, which may disable the establishment of topography (Cheng et al., 2021).

### Inhibitory Synapse Distribution

Synaptic puncta are also distributed in a gradient across the MNTB mediolateral axis. Glycine transporter 2 (GLYT2) positive puncta can be detected in the MNTB prior to hearing onset and increase in expression across the mediolateral axis in the adult mouse (Friauf et al., 1999; Altieri et al., 2014; Milinkeviciute et al., 2021a). Loss of the microglial fractalkine receptor *Cx3cr1*, an ASD and SZ-linked gene (Ishizuka et al., 2017), disrupts the topographic distribution of GLYT2 in the MNTB, leads to a loss of MNTB neural size gradients and faster signal transmission, as measured by the auditory brainstem response (ABR) (Ishizuka et al., 2017; Milinkeviciute et al., 2021a). The MNTB in *Fmr1* KO mice shows elevated levels of GABA/glycinergic marker vesicular GABA transporter (VGAT) (Rotschafer et al., 2015; Ruby et al., 2015). Functionally *Fmr1* KO mice have diminished peak amplitudes as measured by the ABR, and fewer all-or-none EPSCs in the MNTB (Rotschafer et al., 2015; Lu, 2019). Together, these studies show that autism-linked genes appear to influence distributions of ion channels and levels of inhibitory synapses, potentially altering balance in E/I neurotransmission.

## Glial Mechanisms in Synaptic Pruning

Synaptic development, elimination, and maintenance are also mediated by glial cells. *Post-mortem* examinations of ASD or SZ brains have shown increased glial cell number and activation levels, and models of these neurodevelopmental disorders display altered glial pathology (Rodriguez and Kern, 2011; Laskaris et al., 2016). Glia contact and modulate synapses both during development and in the mature brain. Developmental fate-mapping studies show that glial cell expansion during development coincides with the formation of neural circuits in the auditory brainstem; SOC cell-type specific markers co-labeled with glial cell markers in the MNTB (Brandebura et al., 2018). At P0, glia, including microglia, astrocytes, and oligodendrocytes, sparsely occupy lateral regions of the brainstem including the CN and over the first two postnatal weeks can be detected in more medial regions (Dinh et al., 2014; Saliu et al., 2014). In the MNTB, neuron-enriched genes decrease across the first two postnatal weeks, while glia-enriched genes increase within the same time frame (Kolson et al., 2016a). Glial cells interact with calyces and MNTB principal cells in an orchestrated manner both during development and adulthood (Dinh et al., 2014; Kolson et al., 2016a).

### Astrocytes Contact and Modulate MNTB Synapses

Astrocytes contact pre- and postsynaptic membranes in MNTB (Elezgarai et al., 2001) and these contacts elicit slow inward currents through gliotransmisson in the mature MNTB (Reyes-Haro et al., 2010). Astrocytes lie in close apposition to the developing calyx of Held (Figure 1B; Dinh et al., 2014). In the MNTB astrocytes are coupled via gap junctions, and a single astrocyte can contact multiple MNTB principal cells and directly contact calyceal membranes in the active zones of the synapse (Müller et al., 2009; Reyes-Haro et al., 2010). Astrocytes express glutamate transporters and receptors, but calyceal activity does not trigger glutamate uptake currents in astrocytes (Bergles and Jahr, 1997; Renden et al., 2005; Reyes-Haro et al., 2010). The vellous processes of astrocytes contain metabotropic glutamate receptors in mice, reported at P6-18, which allows for uptake of excess glutamate from calyces and prevention of glutamate receptor saturation in the immature calyx (Figure 1B; Elezgarai et al., 2001; Sätzler et al., 2002; Renden et al., 2005; Uwechue et al., 2012).

Pharmacological ablation of microglia during development decreases expression of glial fibrillary acidic protein (GFAP), a marker for mature astrocytes, in the MNTB (Milinkeviciute et al., 2019). When microglia return to control levels following the cessation of treatment, GFAP expression is restored to that of age-matched control mice (Milinkeviciute et al., 2021b). Deletion of Cx3cr1, expressed primarily on microglia, leads to an increase in GFAP expression in the MNTB (Figure 1B; Milinkeviciute et al., 2021a). Mice lacking *Fmr1* have significantly more astrocytes in the VCN and LSO at P14, but there were no differences in the MNTB at this age, despite the abnormal cell numbers and sizes found in the MNTB at that age (Rotschafer et al., 2015). Studies of astrocytes in the auditory brainstem of SZ or ADHD are lacking, despite the evidence of reactive astrogliosis found in SZ or abnormal astrocytosis in ADHD models (Lim and Mah, 2015; Tarasov et al., 2020).

### Oligodendrocytes Regulate Calyx Function

Non-calyceal spaces surrounding MNTB principal cells are filled with microglia, astrocytes, and/or oligodendrocytes (Figure 1B; Elezgarai et al., 2001; Reyes-Haro et al., 2010; Holcomb et al., 2013; Dinh et al., 2014). Firing patterns of GBCs can influence axon diameter and myelin thickness, suggesting that GBCs may regulate their own myelination (Sinclair et al., 2017). Neuron/glia antigen 2 (NG2)-glia, typically regarded as oligodendrocyte progenitor cells (Eugenín-von Bernhardi and Dimou, 2016), also interact with the calyx of Held and receive excitatory input from calyces through AMPA-receptor mediated “synapse-like” inputs (Figure 1B; Müller et al., 2009). NG2 cells are involved in fast signaling with synapses in the mature and developing CNS (Bergles et al., 2000). In the brainstem, oligodendrocytes release BDNF to modulate the glutamate vesicle pool at the nerve terminal, thereby mediating calyx strength and synaptic plasticity in an activity-dependent manner (Berret et al., 2017; Jang et al., 2019). BDNF has been detected at abnormal levels in ASD and SZ patients (Bryn et al., 2015; Gören, 2016; Saghazadeh and Rezaei, 2017; Peng et al., 2018). The development of oligodendrocytes and NG2 cells are dependent on postnatal microglia (Hagemeyer et al., 2017). Although auditory brainstem functions of BDNF in ASD or SZ models are unknown, this signaling pathway could be a regulatory mechanism for E/I balance at the level of the MNTB.

### Microglia Regulate Synapse Elimination and Brainstem Function

**FIGURE 2:**
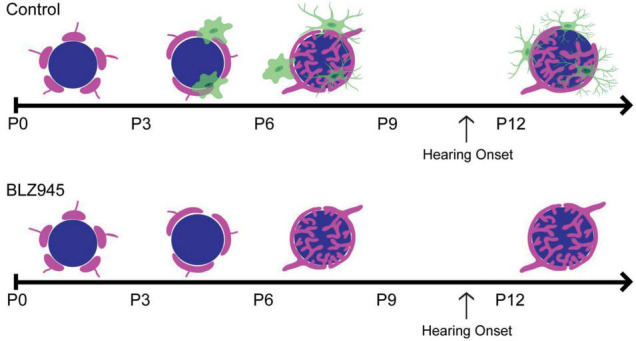
**(Top)** Schematic representation of calyceal pruning during the first two postnatal weeks. Proto-calyces (magenta) innervate MNTB cells (blue) and are eliminated until a single dominant input remains. Microglia (green) are seen near calyces and display mature morphology after hearing onset. **(Bottom)** Microglia elimination with BLZ945 impairs calyceal pruning and leads to higher levels of polyinnervated MNTB neurons. Adapted from Milinkeviciute et al. (2019).

Microglia have increasingly become regarded as circuit sculptors in that they shape axonal projections, eliminate excess synapses, and strengthen intact connections (Paolicelli et al., 2011; Kettenmann et al., 2013). *Post-mortem* examinations of ASD brains showed higher microglial densities in the cerebral cortex (Tetreault et al., 2012) and abnormal microglial-neural spatial organization in the prefrontal cortex (Morgan et al., 2012). Further, inhibition of microglial activation is a potential therapeutic strategy for SZ, and microglial modulation may be a strategy to induce synaptic pruning in ASD (Monji et al., 2009; Andoh et al., 2019). Thus, it is interesting to identify the roles of microglia in auditory circuit development. Microglia can be sparsely detected in the VCN as early as P0, with expression patterns in the MNTB appearing by P6 (Dinh et al., 2014). Microglia in the early postnatal period first appear to have an amoeboid shape and later show a ramified morphology with more extended processes, indicating microglial maturation. In the mouse MNTB, microglial numbers peak by P14, an age after hearing onset. Microglia are in close apposition with the calyx of Held, with their processes interposed between calyces and MNTB principal cells, and peak in number at a time when excess synaptic contacts are pruned (Figure 2; Holcomb et al., 2013; Dinh et al., 2014). Loss of microglia during development impairs calyceal pruning after hearing onset (Figure 2; Milinkeviciute et al., 2019). VGLUT1/2 puncta were observed within microglia, possibly indicating that microglia engulf glutamatergic terminals during pruning. After cessation of the microglial-inhibiting drug BLZ945, microglia gradually returned from lateral to medial regions of the brainstem, recapitulating the pattern seen in normal development. The return of microglia was associated with recovery of auditory brainstem maturation and partial recovery of deficits in the auditory brainstem response (Dinh et al., 2014; Milinkeviciute et al., 2021b). Microglia may also influence the pruning of inhibitory synapses in the auditory system, as deletion of microglial *Cx3cr1* was associated with impaired pruning of inhibitory synapses in MNTB (Milinkeviciute et al., 2021a).

In animals deafened after the first postnatal week, microglia in VCN have more active morphology compared to the control group, which showed more ramified processes (Noda et al., 2019). In mice with cochlear removals activated microglia in the VCN were in close apposition to glutamatergic but not GABAergic synapses (Janz and Illing, 2014). Further, deafening led to an upregulation of phagocytic and anti-inflammatory markers in the VCN (Noda et al., 2019). From these studies, it appears that microglia regulate the elimination of synapses during auditory circuit development. Whether similar findings would be detected in a model of sensory processing disorders is not known. A potential clue is that mice that lack certain autism-linked genes, such as *Fmr1* and *Cx3cr1*, show impaired pruning and that ASD and SZ are linked with abnormal microglia.

## Concluding Remarks

In this review, we discussed factors that are required for normal MNTB development as well as ASD-related models that impair auditory development. The establishment of the MNTB requires factors that regulate axon guidance, development of synapses as well as topographic gradients, synapse elimination, and synapse strengthening. In models of ASD, loss of *Fmr1*, *Pak1*, or *Cx3cr1* results in structural and functional alterations of synapses and an altered glial cell profile. These factors may similarly alter synaptic balance in SZ, ADHD, or other neurodevelopmental disorders.

## Author Contributions

SC, GM, and KC wrote and edited the manuscript. All authors contributed to the article and approved the submitted version.

## Conflict of Interest

The authors declare that the research was conducted in the absence of any commercial or financial relationships that could be construed as a potential conflict of interest.

## Publisher’s Note

All claims expressed in this article are solely those of the authors and do not necessarily represent those of their affiliated organizations, or those of the publisher, the editors and the reviewers. Any product that may be evaluated in this article, or claim that may be made by its manufacturer, is not guaranteed or endorsed by the publisher.
